# Halophilic and halotolerant fungi across diverse climates: a comparative study of Polish and Italian soil ecosystems

**DOI:** 10.3389/fmicb.2025.1637496

**Published:** 2025-07-28

**Authors:** Weronika Śliżewska, Katarzyna Struszczyk-Świta, Anna Otlewska, Flavia Pinzari, Loredana Canfora, Katarzyna Dybka-Stȩpień, Rosario Napoli, Melania Migliore, Andrea Manfredini, Olga Marchut-Mikołajczyk

**Affiliations:** ^1^Institute of Molecular and Industrial Biotechnology, Faculty of Biotechnology and Food Sciences, Lodz University of Technology, Lodz, Poland; ^2^Institute of Fermentation Technology and Microbiology, Faculty of Biotechnology and Food Sciences, Lodz University of Technology, Lodz, Poland; ^3^National Research Council of Italy (CNR), Institute for Biological Systems, Rome, Italy; ^4^Natural History Museum, London, United Kingdom; ^5^Research Centre for Agriculture and Environment (CREA-AA), Council for Agricultural Research and Economics, Rome, Italy

**Keywords:** halophiles, filamentous fungi, salt tolerance, soil microorganisms, fungal diversity, enzymes

## Abstract

This study investigated agricultural saline soils collected from distinct pedoclimatic profiles from Poland and Italy. Twelve fungal strains from Italy and 9 from Poland were identified and tested for halotolerance, extracellular enzyme production, biosurfactant potential, and mycotoxin production. The tested strains were affiliated to 8 genera, with *Aspergillus* and *Penicillium* being the most predominant. All tested strains were confirmed to be good producers of at least one of the analyzed hydrolytic enzymes, with cellulase being the most frequently produced. Notably, *Ramularia mali* FF1 was the best producer of the tested extracellular enzymes and showed the highest enzymatic activity for amylase, cellulase, chitosanase, pectinase and xylanase among all strains. A hemolytic assay was implemented to evaluate the potential for biosurfactant production in media supplemented with various sodium chloride concentrations. Among 21 tested strains, 14 demonstrated hemolytic activity at 5% NaCl. Based on the results, *Acremonium sclerotigenum* FF3 was selected to perform biosurfactant analysis. Mycotoxin screening revealed that *Penicillium canescens* S10 was the only producer of any examined mycotoxin, with 5.759 μg/mL concentration of ochratoxin A. This research underscores the varied enzymatic and biosurfactant capabilities of halophilic fungi adapted to saline soils and highlight the biotechnological potential of these organisms and environments.

## 1 Introduction

Soil salinization is an environmental and economic problem worldwide. This is due to rising sea levels or lithological factors resulting from the weathering and dissolution of rocks containing salts and minerals (primary salinization) or induced by human activities as the use of low-quality water for agricultural purposes and overuse of fertilizers (secondary salinization) (Otlewska et al., 2020; Borges et al., 2023). It is estimated that around the world, up to 20% of total cultivated lands and over 30% of irrigated agricultural lands are saline soils, and this percentage may increase by as much as 10% each year (Shrivastava and Kumar, 2015). The microbial community is also affected by soil salinization. Osmotic and ionic stress, low water activity and alkaline conditions induced by saline soils are often limiting factors for microbial growth, which, in turn, impacts the local microbial diversity (Hou et al., 2021; Ji et al., 2023).

Organisms that can survive and grow in unfavorable conditions of high salinity are called halophiles or halotolerant (Kanekar et al., 2012). Microorganisms from Archaea, Bacteria and Eukarya domains have been found forming structured communities in highly saline environments (Gunde-Cimerman et al., 2004). Simplifying a decidedly varied and nuanced reality, and for mere practicality, one can adopt the Kushner and Kamekura (1988) classification to categorize halophilic microorganisms, which divides microorganisms into groups based on optimal growth at specific concentrations of sodium chloride. Microorganisms that grow optimally in salt concentrations below 1% (0.2 M) NaCl are called **non-halophilic**, however, if they can grow in higher NaCl concentrations (at least 10%), then they are defined as **halotolerant** (Oren, 2008). Among the halophilic organisms, we distinguish **slight halophiles** with optimal growth in 1-3% (0.2–0.5 M) NaCl, **moderate halophiles** growing optimally in the range of 3 to 15% (0.5–2.5 M) NaCl, and **extreme halophiles** with optimal growth in NaCl concentrations above 15% (2.5 M) to even 30% (5.2 M) (Kushner and Kamekura, 1988; Ventosa et al., 2012).

Hypersaline environments present a unique area of study, as they are home to a variety of species that have yet to be thoroughly studied. It is primarily because of the difficulty in isolating them due to the peculiar physiological demands of these organisms, and it is not uncommon to find species in these environments that are new to science, as demonstrated for taxa such as fungi Ascomycota and Basidiomycota (Gunde-Cimerman et al., 2009; Li et al., 2019). Studies indicate that fungi more frequently isolated from saline soils belong to the genera *Aspergillus, Penicillium, Fusarium, Alternaria, Chaetomium*, and *Cephaliophora* (Moubasher et al., 1990; Khodair et al., 1991; Mandeel, 2006; Li et al., 2019; Ji et al., 2023).

Enzymes produced by halotolerant microorganisms are characterized by the fact that their maximum activity is not dependent on the presence or absence of salt in the reaction medium, as may be the case with obligately halophilic enzymes (Delgado-García et al., 2012). These enzymes are also very often polyextremophilic, with greater tolerance over a wide range of temperatures and pH, and function at low water activity and in the presence of organic solvents (Delgado-García et al., 2012; Demirci et al., 2021). Halophilic enzymes usually differ from their non-halophilic counterparts by a greater number of acidic amino acids, with a low proportion of large non-polar and hydrophobic amino acids on the protein surface (Mevarech et al., 2000; Flores-Gallegos et al., 2018). Due to their specific structure and resulting negative charge, halophilic enzymes can maintain their functional conformation, remain soluble, be able to reduce surface hydrophobicity and prevent aggregation at elevated salt concentrations (Graziano and Merlino, 2014; Sinha and Khare, 2014; Lenton et al., 2016). Such salt-tolerant enzymes can play an important role in many industrial processes that require aseptic conditions or special features to overcome high concentrations of NaCl, low water activity or other harsh conditions. Industrial applications of halophilic biomolecules include food industry, agriculture, pharmaceuticals, cosmetics and detergent industry, biofuel production, bioremediation and wastewater treatment etc. (Musa et al., 2018; Akanbi et al., 2020; Chettri et al., 2021; Dutta and Bandopadhyay, 2022; Martínez et al., 2022).

In this study, we aimed to investigate the differences in fungal diversity between saline soils with distinct pedoclimatic profiles from Poland and Italy. Both soils are used for agriculture, and their salinity is due to the presence of salt water from subsurface sources. However, the salinity in Poland originates from the soil itself, while in Italy, it is due to proximity to the sea. Additionally, these two regions experience different climates. This study explored the expected differences and potential overlaps in limiting factors. Therefore, the fungal strains were identified by molecular methods and compared phylogenetically. The assessment included evaluating the halotolerance of the strains, as well as their ability to produce extracellular enzymes and biosurfactants, highlighting their industrial application potential. Additionally, mycotoxin production was examined to ensure the safety of using tested fungal strains in biotechnological and environmental applications.

## 2 Materials and methods

### 2.1 Sampling site description

The study areas are located in Poland and Italy. The collection of salinity soil samples from sites was completed in both countries in September and October 2019.

In Poland, the study sites are located in Pełczyska in the area of “Słone Łaki w Pełczyskach” which is a Special Area of Conservation within Program Nature 2000 (PLH100029), at an altitude of 125-127 m above sea level. The existence of the Salt Meadows in Pełczyska is the result of the presence of Zechstein salt domes, belonging to the so-called Central Polish region of salt domes, and the soil salinization is a result of the presence of mineral spring, which flows from a borehole made in brine outflow drilled at the beginning of the twentieth century. Periodic excess of brine flows through a shallow ditch downhill, where salt meadows with halophytes such as *Atriplex* sp. occur (Czapowski and Bukowski, 2012).

**Figure 1:**
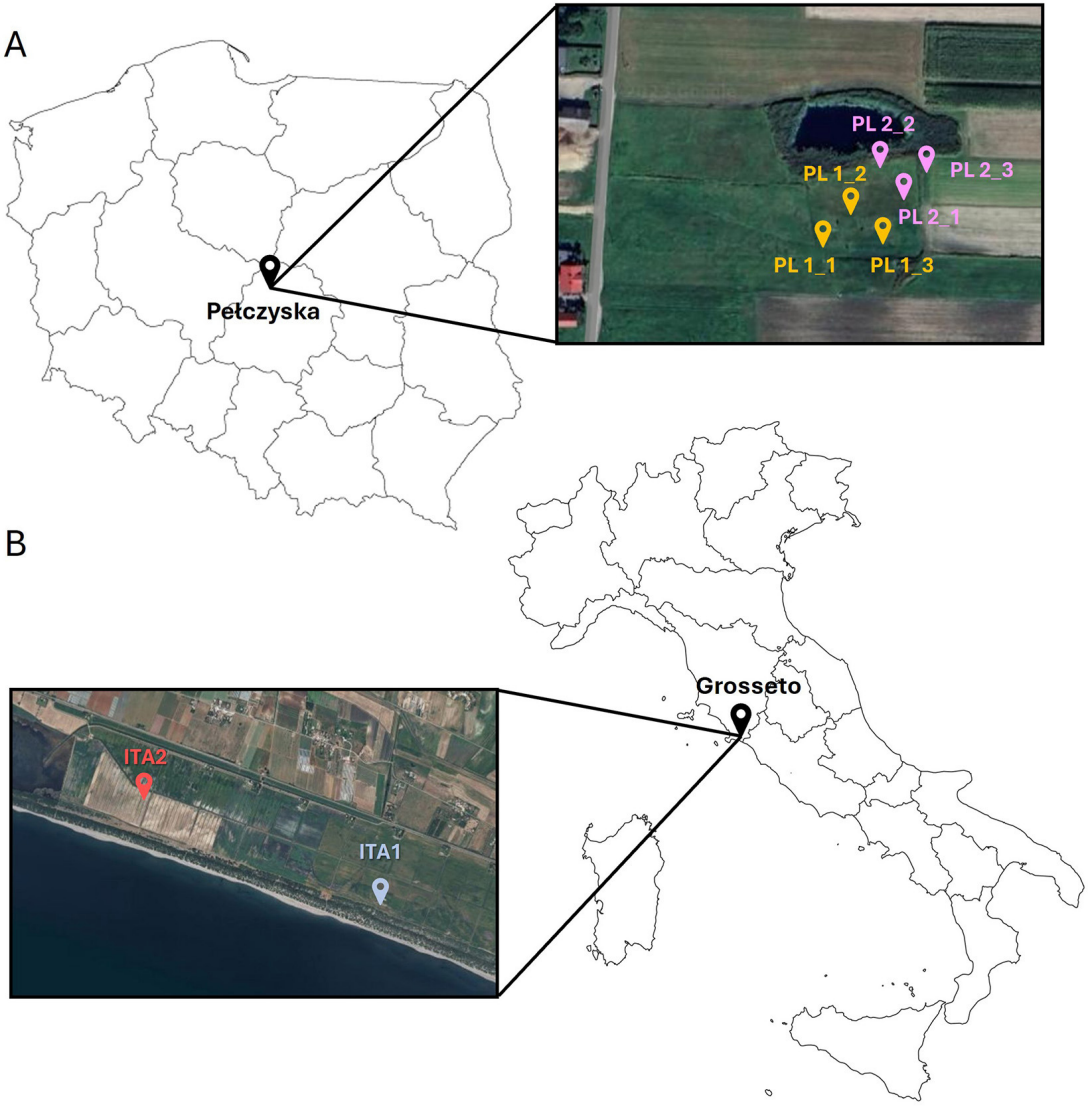
Map of **(A)** Polish and **(B)** Italian isolation area with indicated locations of sampling.

In the Polish field site, samples of the soil were collected in Pełczyska in the Solca Wielka region (51°8′34.08″N, 19°4′20.164″E), 40 km from Łódz, in the north-west part of Łódzkie voivodeship. This sampling site (Figure 1A) was selected based on previous publications which attributed to the area's high concentrations of Na^+^ and Cl^−^ ions (Górecki and Ziułkiewicz, 2016). In the Polish field site of Pełczyska, two areas with contrasting vegetation were selected for soil sampling: one with a high surface salt concentration (PL1) showing the presence of *Atriplex* sp., a plant typically resistant to high salt concentrations and belonging to the family of Chenopodiaceae, and the second (PL2) with entirely different vegetation dominated by *Cynodon* sp., and a slightly different soil profile.

In Italy, the sampling area is located in Grosseto, on the southern coast of the Tyrrhenian Sea, and covered an area located between 0.5 and 0.3 m below sea level. This flat area is mainly constituted by silty, calcareous deposits that filled the retro-lagoonal depressions during the Holocene (D'Orefice et al., 2020). In this area, the salt water table penetrates the depressions and causes a state of natural soil salinization. The soils were non-gravelly, rich in calcium compounds, moderately alkaline, characterized by the presence of decomposed organic deposits on the surface and clay deposits in the deep layers. They have exhibited extreme salinity, along with poor to very poor drainage. Although these soils are considered unsuitable for cultivation, they are used for growing durum wheat.

The Italian field site of the Burano area (Grosseto, Italy) was chosen based on the previous studies carried out by the Italian team from CREA-Agricoltura e Ambiente, who attributed this area's high salinity level (D'Orefice et al., 2020). The study area is located in the Tuscany region (ITA 1−42°23′43.7″N, 11°24′40.0″E; ITA2−42°23′33.8″N, 11°24′49.4″E), in the southern coastal Tyrrhenian border, and consists of soil between 0.5 and 0.3 m below sea level. Two areas (marked as ITA1 and ITA2) with different soil profiles and salinity levels were selected for soil sampling. In this study area, the growth of *Atriplex* sp. was also observed, as in the Polish study area. Samples of the plant were collected for isolation of bacterial and fungal endophytes. Italian sampling sites have been indicated in Figure 1B.

Analysis of soil samples from both Poland and Italy included physicochemical parameters of soils and measurements of soil salinity. From soil samples, fungal isolates were obtained and identified both molecularly and morphologically.

### 2.2 Soil salinity

Soil salinity is a measure of major inorganic salts and minerals dissolved in the water phase of the soil. Those solutes include major cations (e.g. Na^+^, K^+^, Mg^2+^, Ca^2+^) and anions (e.g. Cl^−^, ${\text{HCO}}_{3}^{-}$, ${\text{NO}}_{3}^{-}$, ${\text{SO}}_{4}^{2-}$, ${\text{CO}}_{3}^{2-}$), non-ionic solutes, and combined ions forming ion pairs (Corwin, 2003; Artiola et al., 2019).

Soil salinity was measured indirectly by measuring the electric conductivity (ECe), by passing an electric current between the two electrodes of a salinity meter in aqueous extracts of soil samples (Corwin and Yemoto, 2017).

### 2.3 Fungal isolation

The biodiversity of culturable halophilic soil fungi has been analyzed using classical microbiological methods. A soil sample (10 g) was suspended in 90 mL of saline solution (0.85% NaCl) and gently shaken at 25°C for 1 h. The soil suspension was then sedimented for 30 min at room temperature. The sample was 10-fold diluted in saline, and 100 μL of the suspension was spread onto microbiological media. Petri dishes were incubated at 28°C for up to 14 days to identify halotolerant and halophilic fungi. Four different types of microbiological media were utilized for the fungal analysis: Malt Extract Agar (MEA), Sabouraud (SAB), Czapek-Dox (CYA), and DG18 supplemented with streptomycin. All media were enhanced with salt (NaCl) at four different concentrations: 3%, 5%, 10%, and 15%.

Additionally, a few specimens of *Atriplex* sp. were cleaned using deionized sterile water. The surfaces were disinfected by washing them in 70% ethanol (v/v) for 5 min, followed by treatment with 2% sodium hypochlorite (v/v) for 15 min, and a final wash in 96% ethanol (v/v) for 2 min. This was followed by three rinses with sterile water (Yuan et al., 2010). Segments of tissue were then placed in 8-cm-diameter plates containing Tryptic Soy Agar (TSA), MEA, and Starch Glucose Yeast Agar (SGY), each with three different saline concentrations: 3%, 5%, and 10% NaCl, and were cultured at 25°C. The hyphal tipping technique was employed to purify fungi that developed around the plant tissue segments. Following purification, the fungi were transferred to fresh culture media, which included antibiotics, for further cleaning and identification purposes.

### 2.4 Molecular identification and phylogenetic analysis

All fungal strains were identified based on macroscopic and microscopic morphological features according to diagnostic keys (Bensch et al., 2010, 2012; Gräfenhan et al., 2011; Samson et al., 2014; Videira et al., 2015; Houbraken et al., 2020) and analysis of ITS1/ITS2 nucleotide sequences.

Genomic DNAs were extracted using a Plant and Fungi DNA Purification Kit following the manufacturer's protocol (EURx, Poland). Amplification of the internal transcribed spacer regions (ITS1/ITS2) was performed in MJ Mini Gradient Thermal Cycler (Bio-Rad, Hercules, CA, USA) with universal primer set ITS1 and ITS4 (White et al., 1990). Each PCR reaction was carried out in 50 μL volume containing 40 pmol of each primer, 1.5 U of RedTaq ReadyMix DNA polymerase (Sigma-Aldrich, St. Louis, MO, USA), 20 ng of template DNA and made up to 50 μL with PCR grade water. PCR products were purified and sequenced using Applied Biosystems model 3730 Genetic Analyzer at Genomed S.A. (Warsaw, Poland).

A secondary barcode marker for fungi was used for the Italian strains. Amplification of the translation elongation factor 1-alpha (TEF1-α) was performed with a set of EF1-1018F (forward) and EF1-1620R (reverse) primers according to the method of Stielow et al. (2015). PCR products were purified and sequenced using Sanger dideoxy sequencing. Identification on the Polish strains were confirmed morphologically.

The nucleotide sequences of ITS1/ITS2 regions were proofread, assembled and aligned with nucleotide sequences of ITS/ITS2 region available in The National Center for Biotechnology Information (NCBI, Bethesda, MD, USA) using the blastn algorithm (version BLASTN 2.14.1) and confirmed with UNITE database (Abarenkov et al., 2024). The nucleotide sequences TEF1-α were proofread, assembled and aligned with nucleotide sequences available in The National Center for Biotechnology Information (NCBI, Bethesda, MD, USA) using the blastn algorithm (version BLASTN 2.15.0).

A phylogeny for the isolates was obtained based on ITS sequences, using as reference sequences selected cultured strains downloaded from the NCBI database. Sequence alignment was performed by Clustal W (Thompson et al., 1994). A phylogenetic tree was constructed in MEGA version 11.0.13 (Tamura et al., 2021) using a neighbor-joining method with a bootstrap test of 1,000 replications.

### 2.5 Halotolerance test

The halotolerance test was tested on Malt Extract Agar medium (g/L): malt extract, 30; peptone, 5; agar, 15; pH 5.4, supplemented with a graded series of NaCl concentrations (0%−20%; with an interval of 2.5). Cultures were incubated at 30°C for up to 13 days. The maximum level at which growth occurred was determined as optimal saline conditions (Chamekh et al., 2019).

### 2.6 Enzymatic characterization of fungi

Extracellular enzyme production was evaluated on a solid medium for amylase, cellulase, chitinase, chitosanase, esterase, lipase, protease, pectinase and xylanase. The ability to produce these enzymes was tested on culture media supplemented with 5% sodium chloride with the addition of a specific substrate as a carbon source. Petri dishes with solid medium were inoculated at three points, and the samples were incubated at 30°C for 2–6 days, depending on the strains growth rate. After incubation, colony size and zones of clearing or precipitation around the colony were measured to indicate enzyme production. In some cases, dyes were used to visualize the halo. The results were performed in triplicate. Enzymatic activity was determined by enzymatic index (EI) expressed as EI=R/r, where R is the diameter of the halo and r is the diameter of the colony. Strains that obtained EI equal to or >2 were considered good producers of the tested enzymes for the substrates used (Abe et al., 2015).

The amylolytic activity was detected on nutrient agar medium (g/L): yeast extract, 3; peptone, 5; agar, 20; NaCl, 50; with soluble starch, 2. After incubation, the plates were flooded with twice-diluted Lugol's solution until clear zones appeared around the colonies, indicating starch hydrolysis (Abe et al., 2015).

The cellulolytic activity was determined on solid medium containing (g/L): KH_2_PO_4_, 7; K_2_HPO_4_, 2; MgSO_4_·7H_2_O, 0.1; NH_4_SO_4_, 1; yeast extract, 0.6; agar, 20; NaCl, 50; microcrystalline cellulose, 10. After incubation, the plates were placed at 50°C for 16 h to accelerate the action of extracellular cellulases. To evaluate the clear zone, the plates were flooded with twice diluted Lugol's solution for 5 min (Kasana et al., 2008; Abe et al., 2015).

The chitinolytic activity was screened using modified medium presented by Hankin and Anagnostakis (1975) and Hankin and Anagnostakis (1975). Colloidal chitin prepared by the method described by Kaczmarek et al. (2021) was added in the concentration of 1 g/L to solid medium containing (g/L): MgSO_4_·7H_2_O, 0.1; CaCl_2_, 0.01; KH_2_PO_4_, 0.5; NH_4_NO_3_, 0.5; FeCl_3_, 0.03; yeast extract, 0.2; agar 20; NaCl, 50. After incubation, the plates were flooded with 0.1% Congo red solution for 15 min and then with 1 M NaCl for the same duration to visualize the halo around the colonies.

The chitosanolytic activity was tested on Czapek-Dox solid medium (g/L): with addition of NaCl (5 g/L) and 0.25% (w/v) chitosan as a source of carbon (Shimosaka et al., 1993). 0.5% (w/v) chitosan solution in 2 % (v/v) acetic acid was prepared by the method described by Kaczmarek et al. (2021). After incubation, the plates were flooded with 0.1% Congo red solution for 15 min and then with 1 M NaCl for the same duration in order to visualize the halo around the colonies.

The esterolytic activity was detected on medium containing (g/L): peptone, 10; CaCl_2_·2H_2_O, 0.1; agar, 20; NaCl, 50; and tributyrin (20 mL/L). The tributyrin was autoclaved separately, added to sterile media, homogenized for 5 min and poured onto the plates. Clear zones around colonies indicated esterolytic activity (Molitor et al., 2020).

The lipolytic activity was screened on culture media containing (g/L): peptone, 10; CaCl_2_·2H_2_O, 0.1; agar, 20; NaCl, 50; and Tween 20 (10 mL/L) as a lipid source. The Tween 20 was autoclaved separately and added to sterile media before it was poured on the plates. After incubation, the plates were transferred to 4°C for at least 12 h to better visualize the opaque precipitation around the colonies (Abe et al., 2015).

The proteolytic activity was detected on skim milk agar medium containing skim milk, 300 mL/L; agar, 20 g/L; NaCl, 50 g/L. Clear zones around colonies indicated hydrolysis of casein (Abe et al., 2015).

The pectinolytic activity was screened on medium containing (g/L): MgSO_4_·7H_2_O, 0.1; CaCl_2_, 0.01; KH_2_PO_4_, 0.5; NH_4_NO_3_, 0.5; FeCl_3_, 0.03; yeast extract, 1; agar, 20; NaCl, 50; apple pectin; 5. After incubation, the plates were flooded with 0.1% Congo red solution for 15 min and then with 1 M NaCl for the same duration to visualize the halo around the colonies (Hankin and Anagnostakis, 1975).

The xylanolytic activity was tested on solid medium containing (g/L): yeast extract, 2; peptone, 5; MgSO_4_·7H_2_O, 0.5; CaCl_2_·2H_2_O, 0.1; agar, 20; NaCl, 50; xylan from birch tree, 10. After incubation, the plates were flooded with 0.1% Congo red solution for 15 min and then with 1 M NaCl for the same duration to visualize the halo around the colonies (Babavalian et al., 2013).

### 2.7 Screening for potential biosurfactant production

The potential producers of biosurfactants were screened by testing hemolytic activity (Kiran et al., 2009) on 5% (v/v) sheep blood agar plates containing (g/L): peptone, 5; yeast extract, 3; agar, 15. Medium was also supplemented with 5, 10 or 15% (w/v) NaCl. Plates were incubated at 30°C for 48–96 h (depending on sodium chloride concentration). Clear zones around colonies indicated hemolytic activity which is considered associated with biosurfactant production (Carrillo et al., 1996).

According to the obtained results, the strains with the highest hemolytic activity were selected for further testing. Biosurfactants were produced in liquid Malt Extract Broth (MEB) supplemented with NaCl according to the obtained results of hemolytic activity. A total of 6 flasks of 50 mL of medium were incubated for 7 days on a rotary shaker (180 rpm) at 30°C. The culture broth was centrifuged (18,000 rpm, 4°C, 15 min). The collected supernatant was acidified with 6 M HCl to pH 2 and left overnight at 4°C. The liquid was centrifuged again under the above-mentioned conditions, the supernatant was discarded. The precipitate was dissolved in 0.1 M NaHCO_3_ and lyophilized (Marchut-Mikołajczyk et al., 2021).

The obtained extracted biosurfactants were weighed, and the biosurfactant production efficiency was calculated by dividing the obtained mass of the dried product by the total volume of the medium. In order to determine the emulsifying properties of the obtained biosurfactant, emulsifying activity (OD500) and emulsion index (IE24) were tested. Emulsifying activity was defined according to Pearce and Kinsella's method (Pearce and Kinsella, 1978). 3 mL of 10 μg/mL biosurfactant solution was added to 1 mL phosphate buffer pH 7 and 1 mL diesel fuel. The mixture was homogenized for 30 seconds and then 0.1 mL was transferred to 1 mL of 0.1 % (w/v) sodium dodecyl sulfate (SDS). The absorbance of emulsion was measured in a spectrophotometer at 500 nm. For emulsion index, 2 mL of 10 mg/mL biosurfactant solution was vortexed for 5 min with 2 mL diesel fuel and left at room temperature for 24 h. IE24 was calculated as the height of the emulsion phase divided by the height of the entire mixture and expressed as a percentage.

### 2.8 Detection of mycotoxins

Fungi were cultured in MEB supplemented with 5% (w/v) NaCl at 30°C for 6 days at 120 rpm. After incubation, biomass and supernatant were separated on filtration funnel or centrifuged (10,000 rpm, 10 min, 4°C). For further studies, supernatants were collected and filtrated using syringe filters (0.22 μm pores size, Millex-GS, Millipore, Bedford, MA, USA).

Mycotoxin standards (Sigma-Aldrich, St Louis, MO, USA) of aflatoxin B1 (AFB1), ochratoxin A (OTA), zearalenone (ZEN), deoxynivalenol (DON), and mixture of fumonisin B1 and B2 (FUM) were suspended in PBS buffer (Calbiochem^®^, Germany) to achieve a final concentration 100 μg/mL.

High-performance liquid chromatography (HPLC) analysis of the mycotoxin determination was performed as previously described by Chlebicz and Śliżewska (2020) and Markowiak et al. (2019). To carry out HPLC analysis, the prepared samples were subjected to the Surveyor apparatus (Thermo Scientific, Waltham, MA, USA) with a fluorescent detector (Finnigan Surveyor FL Plus, Thermo Scientific) on Ace 5 C18 column (250 mm × 4.6 mm; Advanced Chromatography Technologies (ACT), Scotland). The parameters of the analysis are presented in Table 1. Mycotoxins were identified by comparing peak retention times with standard solutions, while their concentrations were determined by correlation of sample peak areas with standard curves obtained from mycotoxin standard solutions.

**Table 1:** HPLC analysis parameters for detection of mycotoxins.

**Parameter**	**Mycotoxin**				
	**AFB1**	**OTA**	**ZEN**	**DON**	**FUM**
Column heating	–	–	–	30°C	–
Mobile phase	Water: acetonitrile: methanol (60:30:10)	Water: acetonitrile: acetic acid (99:99:2)	Methanol: water (70:30)	Water: acetonitrile (90:10)	Gradient methanol: water (70:30 and 80:20)
Fluorescent detector λ (excitation and emission) [nm]	360 and 420	330 and 460	280 and 460	–	490 and 450
UV detector λ [nm]	–	–	–	218	–
Flow [mL/min]	1	1	1	1	1

### 2.9 Statistical analyses

Three independent experiments were performed for each sample and means with standard deviations were calculated from the data. Statistical differences between the obtained enzymatic activity, halotolerance results, classification and origin of the isolated strains were compared using a one-way repeated measures analysis of variance (ANOVA; OriginLab Corporation, Northampton, MA, USA). The principal components analysis (PCA) was performed using XLSTAT 2017 (Addinsoft, New York, NY, USA), a statistical add-in for Microsoft Excel (Microsoft, Washington, WA, USA).

## 3 Results

### 3.1 Soil analysis

The results of the physicochemical analyses and characterization of soils presented in Table 2 show the mean salinity values in soil samples from both countries (PL, Poland and ITA, Italy). The values in the higher (S) and lower (C) salinity samples were compared within each site. Means without a common letter (a/b/c) differ significantly (Pr < 0.05), as analyzed by one-way ANOVA. The significant difference between higher and lower salinity samples indicated that the sampling was successful in distinguishing between contrasting areas with different salt content.

**Table 2:** Characterization of sampling sites.

**Sampling area**	**Sampling site**	**Description of soil**	**ECe (dS/m)**	**Pr>F (Model)**
Pełczyska, Poland	PL 1_1	High saline soil (PL_S)	3.46 a	0.004
	PL 1_2			
	PL 1_3			
	PL 2_1	Low saline soil (PL_C)	1.09 b	
	PL 2_2			
	PL 2_3			
Grosseto, Italy	ITA 1_1	Low saline soil (ITA_C)	2.37 b	0.032
	ITA 1_2			
	ITA 1_3			
	ITA 2_1	High saline soil (ITA_S)	8.82 a	
	ITA 2_2			
	ITA 2_3			

### 3.2 Strains identification

The analysis of culturable halophilic soil biodiversity was completed for strains isolated from Italy and Poland using microbiological methods. Twelve isolates from Italian and nine from Polish isolation areas were selected for molecular identification and further research.

**Figure 2:**
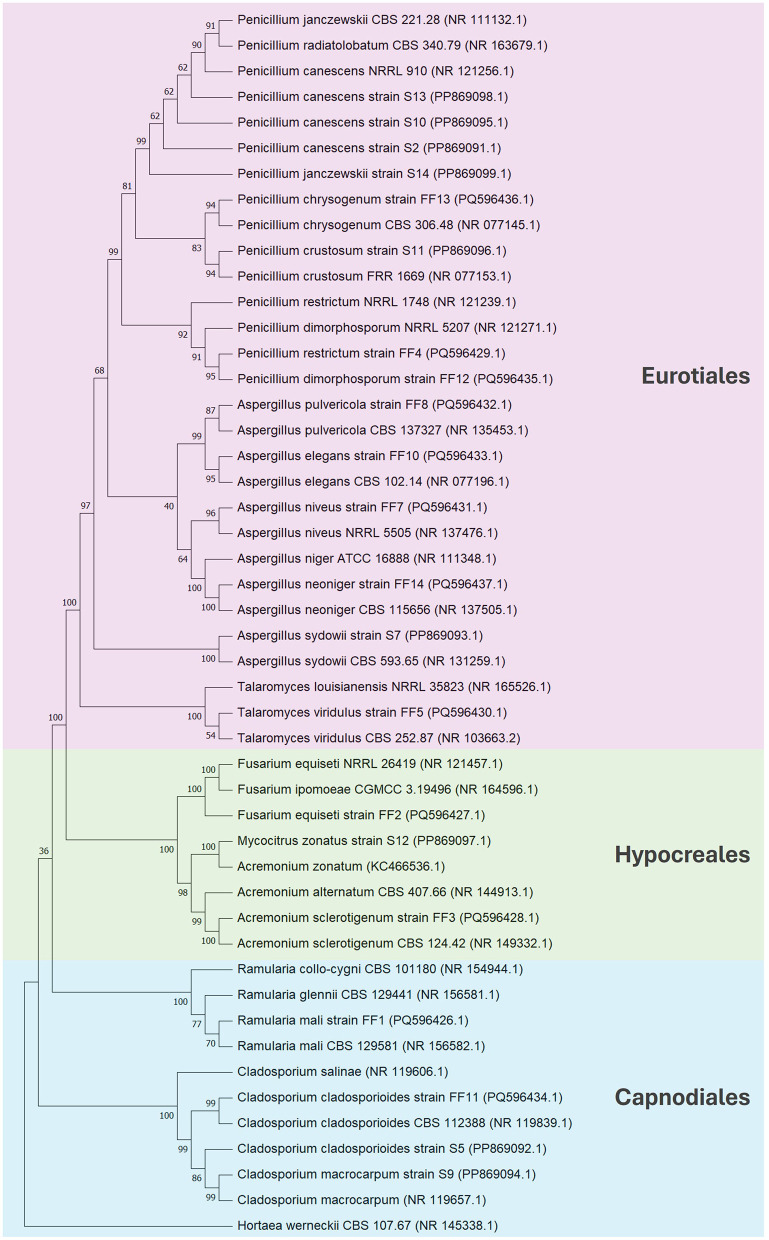
Phylogenetic tree constructed using the neighbor-joining method based on the comparison of ITS sequences. *Hortaea werneckii* CBS 107.67 (NR 145338.1) was used as an outgroup taxon.

The obtained fungal ITS sequences were assembled taxonomically using BLASTN 2.14.1 and deposited in the GenBank database. A list of fungal strains and their molecular identification is shown in Table 3. All the tested stains belonged to Ascomycota division, among which they belonged to orders Euriotiales (14), Capnodiales (4) and Hypocreales (3). *Aspergillus* and *Penicillium* were the most abundant genera in total, while *Aspergillus* dominated Italian soils and *Penicillium* was the most common in Polish samples. Other genera that were found in both environments were *Cladosporium* and *Acremonium (Mycocitrus*). Italian saline soils were characterized by more diverse genera with *Ramularia, Fusarium* and *Talaromyces* which only occurred in this area. Biodiversity of fungal community in both tested zones is shown as phylogenetic tree presented in Figure 2.

**Table 3:** Fungal isolates screened in this study and their molecular identification.

**Strains code**	**Strains identity**	**Order**	**Isolation area**	**Isolation site**	**Source**	**Identity of ITS (UNITE or BLAST)**	**Accession number of ITS**	**Accession number of TEF1-α**
FF1	*Ramularia mali*	Capnodiales	Grosseto, Italy	ITA 1_1	Saline soil	100%	PQ596426	PQ644613
FF2	*Fusarium equiseti*	Hypocreales		R	Root of *Atriplex L*.	100%	PQ596427	PQ644614
FF3	*Acremonium sclerotigenum*	Hypocreales		R		100%	PQ596428	PQ644615
FF4	*Penicillium restrictum*	Eurotiales		ITA 1_3	Saline soil	99.8%	PQ596429	PQ644616
FF5	*Talaromyces viridulus*	Eurotiales		ITA 2_1		98.11%	PQ596430	PQ644617
FF7	*Aspergillus niveus*	Eurotiales		ITA 1_3		98.67%	PQ596431	PQ644618
FF8	*Aspergillus pulvericola*	Eurotiales		ITA 1_2		98.19%	PQ596432	PQ644619
FF10	*Aspergillus elegans*	Eurotiales		ITA 1_2		99.18%	PQ596433	PQ644620
FF11	*Cladosporium cladosporioides*	Capnodiales		ITA 1_1		99.81%	PQ596434	PQ644621
FF12	*Penicillium dimorphosporum*	Eurotiales		ITA 1_3		99.29%	PQ596435	PQ644622
FF13	*Penicillium chrysogenum*	Eurotiales		ITA 1_3		99.64%	PQ596436	PQ644623
FF14	*Aspergillus neoniger*	Eurotiales		ITA 1_1		100%	PQ596437	PQ644624
S2	*Penicillium canescens*	Eurotiales	Pełczyska, Poland	PL 1_3	Saline soil	99.83%	PP869091	
S5	*Cladosporium cladosporioides*	Capnodiales		PL 2_2		99.19%	PP869092	
S7	*Aspergillus sydowii*	Eurotiales		PL 1_3		98.88%	PP869093	
S9	*Cladosporium macrocarpum*	Capnodiales		PL 1_3		99.44%	PP869094	
S10	*Penicillium canescens*	Eurotiales		PL 1_3		99.83%	PP869095	
S11	*Penicillium crustosum*	Eurotiales		PL 2_2		100 %	PP869096	
S12	*Mycocitrus zonatus*	Hypocreales		PL 1_2		99.82%	PP869097	
S13	*Penicillium canescens*	Eurotiales		PL 1_1		100%	PP869098	
S14	*Penicillium janczewskii*	Eurotiales		PL 1_1		99.82%	PP869099	

### 3.3 Halotolerance test

All 21 tested fungal strains were able to grow on MEA medium with different salt concentrations, as shown in Table 4. The optimal growth of most strains was observed for MEA containing 5% NaCl, therefore majority of the strains were classified as moderately halophilic. This group also includes strains with an optimal growth at 7.5% NaCl (*Cladosporium macrocarpum* S9 and *Penicillium canescens* S13). Six strains with optimal growth at 2.5% NaCl were classified as slight halophilic, while three strains (*Acremonium sclerotigenum* FF3, *Penicillium restrictum* FF4, *Talaromyces viridulus* FF5) were considered halotolerant as they grew best in medium without NaCl.

**Table 4:** Salt tolerance and optimal growth salt concentrations of fungal isolates.

**Strains code**	**Strains**	**Salt tolerance (NaCl %)**		
		**Growth range**	**Optimal growth**	**Classification** ^a^
**FF1**	*Ramularia mali*	0–10	5	MH
**FF2**	*Fusarium equiseti*	0–12.5	2.5	SH
**FF3**	*Acremonium sclerotigenum*	0–12.5	0	HT
**FF4**	*Penicillium restrictum*	0–15	0	HT
**FF5**	*Talaromyces viridulus*	0–10	0	HT
**FF7**	*Aspergillus niveus*	0–17.5	5	MH
**FF8**	*Aspergillus pulvericola*	0–15	5	MH
**FF10**	*Aspergillus elegans*	0–17.5	5	MH
**FF11**	*Cladosporium cladosporioides*	0–10	5	MH
**FF12**	*Penicillium dimorphosporum*	0–17.5	5	MH
**FF13**	*Penicillium chrysogenum*	0–17.5	2.5	SH
**FF14**	*Aspergillus neoniger*	0–17.5	2.5	SH
**S2**	*Penicillium canescens*	0–15	5	MH
**S5**	*Cladosporium cladosporioides*	0–12.5	2.5	SH
**S7**	*Aspergillus sydowii*	0–17.5	2.5	SH
**S9**	*Cladosporium macrocarpum*	0–12.5	7.5	MH
**S10**	*Penicillium canescens*	0–15	5	MH
**S11**	*Penicillium crustosum*	0–15	2.5	SH
**S12**	*Mycocitrus zonatus*	0–10	5	MH
**S13**	*Penicillium canescens*	0–15	7.5	MH
**S14**	*Penicillium janczewskii*	0–17.5	5	MH

^a^See for explanation: HT, halotolerant; SH, slightly halophilic; MH, moderately halophilic.

Each strain was able to grow on media supplemented with NaCl ranging from 0 to 10%, and most tolerated even higher salt concentrations up to 17.5%. None of the tested strains was able to grow in 20% NaCl.

### 3.4 Enzymatic characterization of fungi

The strains were tested for the production of nine extracellular enzymes (Table 5) which included amylase, cellulase, chitinase, chitosanase, esterase, lipase, protease, pectinase and xylanase.

**Table 5:** Enzymes produced by halophilic fungi with indication of enzymatic index.

**Strains code**	**Amylolytic activity**	**Cellulolytic activity**	**Chitinolytic activity**	**Chitosanolytic activity**	**Esterolytic activity**	**Lipolytic activity**	**Proteolytic activity**	**Pectinolytic activity**	**Xylanolytic activity**
**FF1**	**5.69** **±0.86**	**7.84** **±0.88**	–	**2.69** **±0.26**	**3.83** **±0.53**	**3.77** **±0.43**	**3.21** **±0.55**	**10.67** **±1.70**	**8.63** **±1.11**
**FF2**	–	**5.99** **±1.00**	–	–	–	–	–	–	–
**FF3**	1.89 ± 0.18	**3.57** **±0.65**	–	1.38 ± 0.09	–	1.74 ± 0.15	1.27 ± 0.07	1.48 ± 0.13	**2.02** **±0.50**
**FF4**	**4.59** **±1.41**	**6.46** **±0.54**	–	1.88 ± 0.31	1.64 ± 0.22	1.90 ± 0.43	**3.45** **±0.49**	**2.89** **±0.39**	**3.41** **±0.69**
**FF5**	1.82 ± 0.22	**5.42** **±0.51**	–	–	–	1.93 ± 0.11	–	1.54 ± 0.08	**3.03** **±0.62**
**FF7**	–	**7.25** **±1.65**	–	–	1.66 ± 0.28	–	–	1.86 ± 0.14	1.53 ± 0.06
**FF8**	1.44 ± 0.11	**4.88** **±0.56**	–	–	1.50 ± 0.25	**2.38** **±0.76**	–	1.29 ± 0.03	1.50 ± 0.11
**FF10**	1.74 ± 0.16	**4.33** **±0.40**	–	–	1.44 ± 0.14	1.95 ± 0.22	1.20 ± 0.06	1.39 ± 0.04	1.54 ± 0.11
**FF11**	**2.00** **±0.27**	**2.77** **±0.22**	–	–	**2.33** **±0.54**	**2.38** **±0.48**	–	**2.83** **±0.34**	**3.53** **±0.24**
**FF12**	**2.35** **±0.59**	**4.40** **±0.58**	–	–	1.26 ± 0.05	1.63 ± 0.16	–	1.63 ± 0.17	1.56 ± 0.14
**FF13**	**2.00** **±0.22**	**4.15** **±0.25**	–	–	1.25 ± 0.07	1.54 ± 0.20	–	1.66 ± 0.27	1.42 ± 0.09
**FF14**	1.89 ± 0.31	**4.37** **±0.57**	–	1.39 ± 0.13	1.86 ± 0.28	1.37 ± 0.06	–	–	1.77 ± 0.25
**S2**	1.34 ± 0.07	**5.58** **±0.62**	–	–	**3.99** **±0.43**	1.89 ± 0.28	1.19 ± 0.04	1.26 ± 0.06	1.52 ± 0.08
**S5**	1.28 ± 0.09	**3.07** **±0.16**	–	–	**2.27** **±0.37**	1.80 ± 0.31	1.44 ± 0.19	1.77 ± 0.39	**4.31** **±0.38**
**S7**	1.87 ± 0.13	**4.13** **±0.65**	–	–	1.21 ± 0.03	1.39 ± 0.06	1.18 ± 0.03	1.68 ± 0.22	1.83 ± 0.13
**S9**	**2.50** **±0.66**	**6.21** **±0.46**	–	–	–	**4.25** **±0.28**	**2.26** **±0.48**	**4.01** **±0.15**	**6.35** **±1.12**
**S10**	1.40 ± 0.06	**6.34** **±0.53**	–	–	**4.38** **±0.47**	1.54 ± 0.15	1.19 ± 0.07	1.37 ± 0.20	1.60 ± 0.20
**S11**	**2.39** **±0.31**	**4.76** **±0.38**	–	1.26 ± 0.05	1.46 ± 0.21	**2.49** **±0.55**	–	1.85 ± 0.22	–
**S12**	–	**5.86** **±0.70**	–	1.35 ± 0.07	–	–	1.12 ± 0.03	–	1.18 ± 0.06
**S13**	**2.18** **±0.30**	**6.09** **±0.97**	–	1.47 ± 0.10	**2.37** **±0.30**	**2.21** **±0.30**	–	**2.20** **±0.30**	1.85 ± 0.38
**S14**	–	**3.26** **±0.59**	–	–	**2.04** **±0.54**	**2.00** **±0.30**	1.68 ± 0.35	**2.03** **±0.19**	–
**Total (21)**	**8**	**21**	**0**	**1**	**7**	**7**	**3**	**6**	**7**

Good producers of tested enzymatic activities (EI ≥ 2) were highlighted.

All strains produced at least one enzyme for which they could be considered good producers (EI ≥ 2). Cellulase was the enzyme most frequently produced by the tested halophilic fungi. Among 17 amylase-producing strains, 8 can be qualified as good producers of this enzyme, and the highest activity was obtained for *Ramularia mali* FF1 with EI = 5.69 and *Penicillium restrictum* FF4 with EI = 4.59. For about 86% of the strains, lipase, pectinase and xylanase were detected, but only 7 (lipase and xylanase) or 6 (pectinase) strains can be considered good producers of these enzymes. The highest lipolytic activity of EI = 4.25 was found in *Cladosporium macrocarpum* S9, while the highest pectinolytic and xylanolytic activities of 10.67 and 8.63 were shown by *R. mali* FF1. Esterolytic activity was confirmed for the 16 tested strains, but only one-third showed an EI above 2, of which the highest activity with EI of 4.38 was obtained for *Penicillium canescens* S10. Protease was produced by 52% of the tested strains, but only *R. mali* FF1*, P. restrictum* FF4 and *C. macrocarpum* S9 showed an EI > 2.0 among which *P. restrictum* FF4 had the highest activity. Seven strains secreted chitosanases, but only *R. mali* FF1 could be considered a good producer of this enzyme. None of the tested strains showed chitinolytic activity.

The best producer of the tested extracellular enzymes was *R. mali* FF1, which produced all enzymes except chitinase, and for amylase, cellulase, chitosanase, pectinase, and xylanase, it showed the highest enzymatic activity among all strains. The strain producing 8 out of 9 tested enzymes was also *P. restrictum* FF4, with an EI > 2.0 for amylase, cellulase, protease, pectinase and xylanase. *C. cladosporioides* FF11 and *C. macrocarpum* S9 were also strains with a wide range of enzyme activities, both producing 6 enzymes with EI ≥ 2.0.

### 3.5 Screening of biosurfactant production

All the halophilic filamentous fungi strains were tested as potential biosurfactant producers. Preliminary screening (Table 6) conducted by hemolytic assay showed that 14 out of the 21 tested strains had at least minimal hemolytic activity on medium containing 5% (w/v) NaCl, but only 4 of them showed also hemolytic activity on medium with 10% (w/v) NaCl. None of the strains showed hemolytic activity on medium supplemented with 15% (w/v) NaCl. *Acremonium sclerotigenum* FF3 was the only strain with a large halo zone around colonies, indicating the biggest hemolytic activity. It is worth noting that this strain showed a greater level of hemolytic activity in media that contained a higher concentration of salt. For this reason, *Acremonium sclerotigenum* FF3 was selected for further analysis.

**Table 6:** Qualitative estimation of hemolytic activity of halophilic fungal strains for potential biosurfactant production.

**Strains code**	**Strains**	**Hemolytic activity**		
		**5% NaCl**	**10% NaCl**	**15% NaCl**
**FF1**	*Ramularia mali*	+	–	–
**FF2**	*Fusarium equiseti*	+	–	–
**FF3**	*Acremonium sclerotigenum*	+	+++	–
**FF4**	*Penicillium restrictum*	–	–	–
**FF5**	*Talaromyces viridulus*	+	–	–
**FF7**	*Aspergillus niveus*	+	+	–
**FF8**	*Aspergillus pulvericola*	++	+	–
**FF10**	*Aspergillus elegans*	++	++	–
**FF11**	*Cladosporium cladosporioides*	–	–	–
**FF12**	*Penicillium dimorphosporum*	–	–	–
**FF13**	*Penicillium chrysogenum*	–	–	–
**FF14**	*Aspergillus neoniger*	–	–	–
**S2**	*Penicillium canescens*	+	–	–
**S5**	*Cladosporium cladosporioides*	+	–	–
**S7**	*Aspergillus sydowii*	–	–	–
**S9**	*Cladosporium macrocarpum*	–	–	–
**S10**	*Penicillium canescens*	++	–	–
**S11**	*Penicillium crustosum*	+	–	–
**S12**	*Mycocitrus zonatus*	+	–	–
**S13**	*Penicillium canescens*	+	–	–
**S14**	*Penicillium janczewskii*	+	–	–

(–) no activity, (+) minimum, (++) medium, (+++) maximum activity.

The biosurfactant compound extracted from *Acremonium sclerotigenum* FF3 was tested for its emulsifying properties. The extraction yield of biosurfactant reached 3.18 g/L. For both emulsifying activity and emulsion index analysis, 10 mg/mL of biosurfactant solution was a starting concentration. In case of emulsion index, no stable emulsion was noticed after 24 h. To obtain measurable results within apparatus range, sample was diluted 500 × to concentration of 10 μg/mL which resulted in OD_500_ 1.426. It was confirmed that *A. sclerotigenum* FF3 is a potential producer of the biosurfactant, and its detailed characterization requires further research.

### 3.6 Detection of mycotoxins

Halophilic fungi were checked for the production of mycotoxins, which include aflatoxin B1 (AFB1), ochratoxin A (OTA), zearalenone (ZEN), deoxynivalenol (DON), and mixture of fumonisin B1 and B2 (FUM). Among all the tested strains, only *Penicillium canescens* S10 was a producer of one of the tested mycotoxins. The concentration of ochratoxin A produced by *P. canescens* S10 was 5.759 μg/mL.

### 3.7 Statistical analysis

Correlation between halotolerance results, enzymatic activities and place of isolation were tested using statistical parameters. The 12 variables were included in statistical analysis: maximum NaCl concentration resulting in growth, optimal growth NaCl concentration, halophilic microorganisms classification according to Kushner and Kamekura (1988), amylolytic activity, cellulolytic activity, chitosanolytic activity, esterolytic activity, lipolytic activity, proteolytic activity, pectinolytic activity, xylanolytic activity and region from which strains were isolated. Chitinolytic activity was excluded from the statistical analysis because none of the tested strains showed this activity.

**Figure 3:**
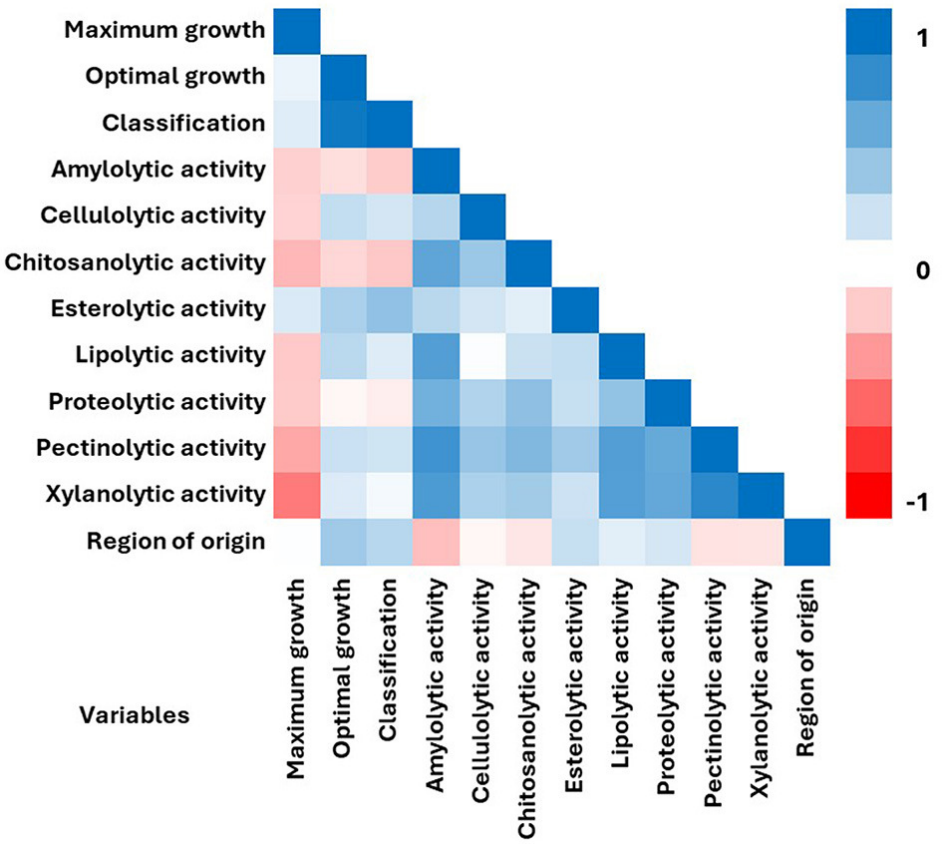
Pearson correlation matrix among the 12 variables including sodium chloride concentration for maximum and optimal growth, halophilic classification, enzymatic activities and region of isolation. Matrix was measured for 21 halophilic fungal strains. The color denotes the correlation between two traits where a complete positive correlation (blue) is represented by 1 and a complete negative correlation (red) is represented by −1.

Correlation analyses were performed to determine the correlation coefficient between parameters related to the features of the tested halophilic strains (Figure 3). Of the 12 features examined, there was a positive correlation between most of the traits. Among all parameters, the highest positive correlation was between optimal growth conditions and classification of strains (*r* = 0.943). The second highest correlation was noted between xylanolytic and pectinolytic activities (*r* = 0.832). Overall, the strongest positive correlations were observed for amylolytic activity while compared with other enzymatic activities: pectinolytic (*r* = 0.757), xylanolytic (*r* = 0.703), lipolytic (*r* = 0.675), chitosanolytic (*r* = 0.630) and proteolytic (*r* = 0.555) activities. The strongest negative correlation was observed for maximum growth NaCl concentrations and xylanolytic activity (*r* = −0.514). Sodium chloride concentration did not show correlation with “maximum” and “optimal” growth variables. The “region of isolation” did not show significant correlations with any of the analyzed traits, and its highest positive correlation was observed only with “optimal growth conditions” (*r* = 0.371). Also, the Pearson correlation coefficients indicated that the “region of isolation” or “halotolerance” variables of the tested strains did not correlate with any enzyme production.

**Figure 4:**
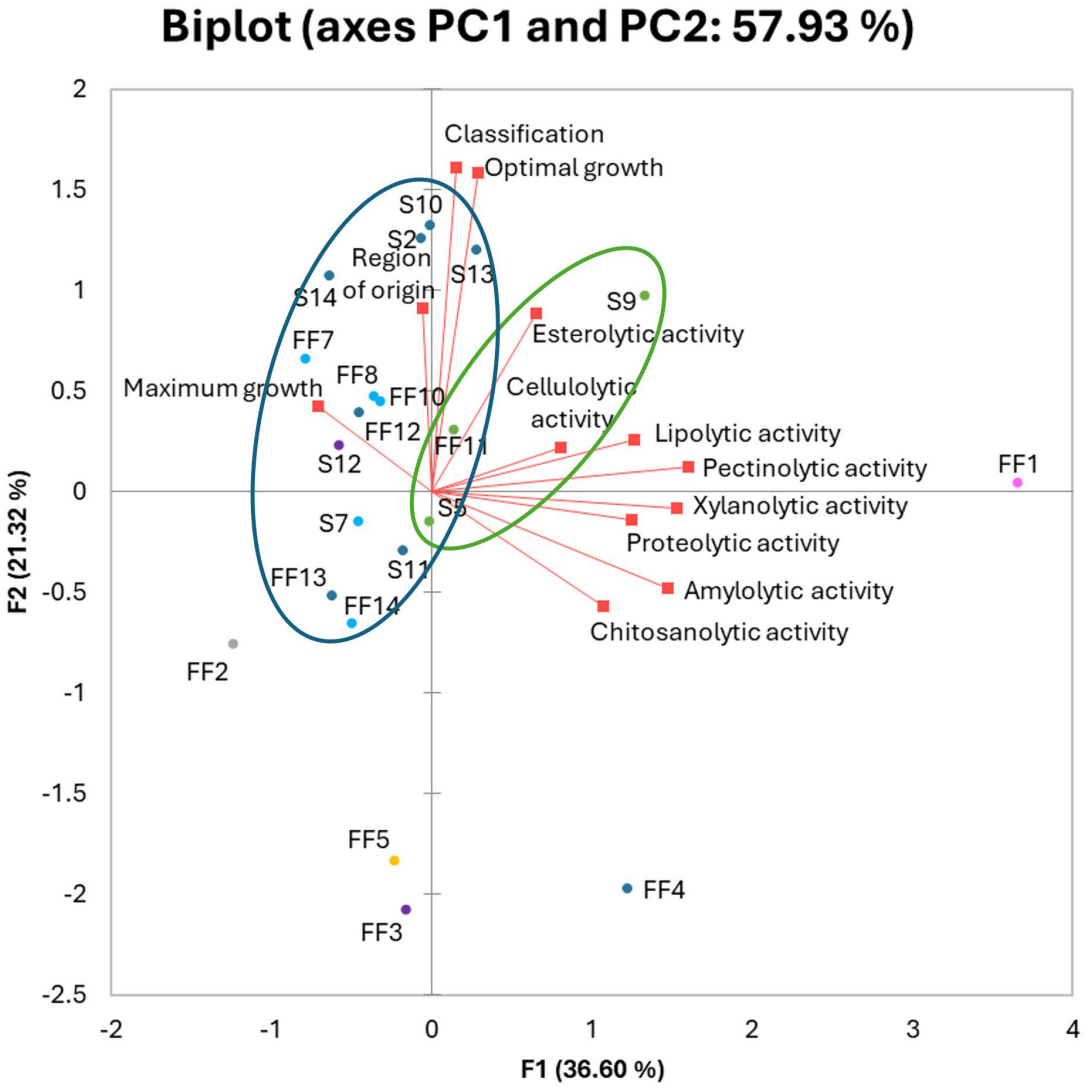
Principal Component Analysis (PCA) biplot of tested halophilic fungal strains. Active variables (red squares). Active observations (circles) were divided based on genus: *Aspergillus* (light blue), *Acremonium* and *Mycocitrus* (purple), *Cladosporium* (green), *Fusarium* (gray), *Penicillium* (dark blue), *Ramularia* (pink), *Talaromyces* (yellow). Strains from FF1–FF14 were isolated from Italy, while strains S2–S14 were obtained from Poland.

A principal component analysis (PCA) was performed on all the traits (Figure 4) to identify the variables better grouping the fungal isolates and any trend in their common ecology. According to the PCA model, the total data variance was 57.93%, which was attributed to PC1 with 36.6% and to PC2 with 21.32%. Among the traits examined, PC1 was strongly correlated with most of enzymatic activities. Meanwhile PC2 was mostly correlated with optimal growth NaCl concentration and halophilic classification, but also region of origin and maximum growth conditions. Esterolytic activity was evenly positively correlated with both PC1 and PC2. Analyzing the distribution of tested strains on the biplot graph, it can be observed that the strains are grouped according to their genus rather than to any other feature. A cluster of strains belonging to *Cladosporium* (green lines) and a second cluster grouping *Penicillium* and *Aspergillus* isolates (blue lines) can be drawn. The *Ramularia mali* (FF1), *Fusarium equiseti* FF2 and *Talaromyces viridulus* FF5 separate from the other strains in the biplot. *Penicillium restrictum* FF4 was the only strain which did not group with rest of *Penicillium* and *Aspergillus* strains. No clustering of strains isolated from the same environment was observed, but Polish strains (S2–S9) were mostly positively correlated with PC2. Both Polish and Italian strains were mostly negatively correlated with PC1.

## 4 Discussion

The present study focused on identifying and determining the enzymatic potential of halophilic filamentous fungi isolated from saline soils in Poland and Italy with contrasting pedoclimatic characteristics.

Saline soils are defined as soils characterized by a high concentration of soluble salts when the electrical conductivity of the saturated paste extract (ECe) is ≥4 dS/m. However, some scientists recommend lowering the ECe limit for saline soils to 2 dS/m, because even such salinity may affect many crops (Sparks, 2003; Munns, 2005). In the sampling fields, the average salinity of agricultural soils was measured as specific conductivity in a saturated soil solution (EC_e_). In Pełczyska (Poland), saline soil samples (PL1) had an average salinity level of 3.46 dS/m, while control samples (PL2) had an average level of 1.09 dS/m. These levels were significantly lower than the levels found in comparable samples taken from Grosseto (Italy), where saline soil samples (ITA1) had a salinity level of 8.82 dS/m and control sample (ITA2) had a level of 2.37 dS/m. On the basis of the results, according to the classification presented by Harper et al. (2021), Polish soils can be classified as slightly saline, while Italian soils as strongly saline.

Despite the differences between the EC_e_ and different classifications of soil salinity, which showed that Italian soils were highly more saline than Polish soils, the halotolerance of isolated fungal strains obtained from both environments were statistically insignificant. Moreover, some strains from both environments showed relatively high salt tolerance, even though isolated from rather low-salinity environments. Fungal strains isolated from the Polish field site showed higher salt concentrations for optimal growth, with 67% being moderately halophilic and 33% slightly halophilic. None of the Polish strains were classified as halotolerant. On the other hand, 50% of the Italian strains were moderately halophilic and 25% were respectively classified as slightly halophilic and halotolerant. Despite the lower NaCl concentrations required for optimal growth, more Italian strains tolerated higher salt concentrations of 17.5% NaCl.

This could be due to origin of salinization of the soils. Polish soils are historically saline, because of the mineral springs and salt domes in the area, therefore their salinity is probably more stable over time. One the other hand, salinity of Italian soils is fluctuating constantly due to their location in coastal area and being flooded regularly by sea waves. Therefore, fungi which accessed a wide spectrum of salt tolerance were more likely to survive. It was already suggested that halotolerant microorganisms isolated from environments with fluctuating salinity like coastal areas or mangrove ecosystems were more resistant to higher salt concentrations (Agwu and Oluwagunke, 2014; Suryanarayanan and Ravishankar, 2023).

Soil fungal communities are primarily composed of Ascomycota (Hou et al., 2021; Ji et al., 2023), and this was also observed in this study. *Aspergillus* and *Penicillium* were identified as the most dominant genera in the analyzed saline soils. These genera have been reported as often occurring in saline environments (Moubasher et al., 1990; Khodair et al., 1991; Gunde-Cimerman and Zalar, 2014; Moubasher et al., 2018). Other genera commonly found in saline conditions that were identified in this study were *Cladosporium* (Cantrell and Baez-fé Lix, 2010; Zajc et al., 2012), *Fusarium* (Mandeel, 2006), *Acremonium* (Cantrell and Baez-fé Lix, 2010) and *Talaromyces* (González-Martínez et al., 2017; Ren et al., 2017). The only genus that was not found earlier in halophilic conditions was *Ramularia* which is known worldwide as a plant pathogenic species causing leaf spotting symptoms (Videira et al., 2015; Bakhshi and Arzanlou, 2017). Meanwhile, *Ramularia mali* FF1 described in this study, isolated from Italian saline soil, was characterized as moderately halophilic for showing optimal growth at 5% NaCl concentration and tolerating up to 10% NaCl addition, making it the first halophilic *Ramularia* strain.

The production of enzymes by microorganisms is a crucial factor that allows their use in various industries (Elleuche et al., 2014). Particularly noteworthy are enzymes produced by extremophilic microorganisms, which include enzymes from halophiles and halotolerant species with increased tolerance to salt and the presence of organic solvents compared to their mesophilic counterparts (Amoozegar et al., 2019; Śliżewska et al., 2022). The ability to produce nine extracellular hydrolases was evaluated for 21 strains of halophilic and halotolerant fungi in the presence of 5 % NaCl. Hydrolytic haloenzymes including: amylase, cellulase, chitinase, chitosanase, esterase, lipase, protease, pectinase, and xylanase were tested. All studied strains were confirmed to produce at least one of the tested hydrolytic enzymes.

The enzyme that was produced by all tested strains and showed the highest activity for most of them was cellulase. This may be related to the fact that cellulose (an organic compound forming the plant cell wall, which is hydrolyzed by cellulase) was widely available in the studied environments, which made it an accessible carbon source for the tested fungi (Sharma et al., 2024). The highest cellulolytic activity was indicated for strains *Ramularia mali* FF1 and *Aspergillus niveus* FF7 with EI of 7.84 and 7.25, respectively. It was previously reported that *A. niveus* was a great producer of cellulolytic enzymes even in thermolytic (Taj-Aldeen and Jaffar, 1992; Taj-Aldeen and Alkenany, 1993) and halophilic conditions (Malik et al., 1980). In the case of *Ramularia* species, it was only demonstrated in the genome that they could be a source of cellulolytic enzymes, but this has not been proven phenotypically (McGrann et al., 2016). Due to the characteristics of enzymes from halophiles, the tested cellulases may find application in the biofuel industry (Yin et al., 2015; Amoozegar et al., 2019).

The second most widely produced enzyme was amylase with highest values of EI = 5.69 for *R. mali* FF1 and EI = 4.59 for *Penicillium restrictum* FF4. Same species had been great producers of protease with EI of 3. Several research showed that *Penicillium* species has been important source of amylolytic (Khokhar et al., 2011; Dendouga and Belhamra, 2022) and proteolytic enzymes (Germano et al., 2003; Devi et al., 2022).

The best producers of pectinolytic and xylanolytic activity were *R. mali* FF1 and *Cladosporium macrocarpum* S9. Although in the case of *Ramularia*, there is no data regarding the enzymatic properties of this species, *C. macrocarpum* was a proven producer of xylanase (Fattah et al., 2011), while several *Cladosporium* sp. were reported for pectinase production (Poveda et al., 2018; Moharram et al., 2021, 2022).

Halophiles are also well-known producers of esterases and lipases (Schreck and Grunden, 2014). For esterases, highest activity was determined for fungi from *Penicillium* species*, P. canescens* S10 (EI 4.38) and *P. canescens* S2 (EI 3.99). Studies have shown that they can be an excellent source of esterase and lipase with various substrate specificity (Li and Zong, 2010; Sahay and Chouhan, 2018). *C. macrocarpum* S9 was the best producer of lipolytic enzymes (EI 4.25), also reported earlier for this genus (Chinaglia et al., 2014; Abe et al., 2015).

Although none of the tested strains produced chitinase, a few strains could be potentially a source of chitosanase. Those strains were *R. mali* FF1, *P. restrictum* FF4, *P. canescens* S13, *A. neoniger* FF14 and *P. crustosum* S11. *Penicillium* and *Aspergillus* have been already known as chitosanase-producing fungi (Fenton and Eveleigh, 1981; Dhillon et al., 2011; Cao et al., 2022; Abedin et al., 2023), however, interestingly only research on *Ramularia* genus shows inhibiting influence of chitosan on the growth with no record of chitosanolytic activity (Quintana-Obregón et al., 2011).

Overall, the fungal strains isolated in this study from saline soils in Poland and Italy distinguished higher enzymatic activities expressed by the Enzymatic Index compared to results reported previously for analog species obtained from both halophilic and non-halophilic species. Similar studies about halophilic fungal strains isolated from Sebkha El Melah (Jaouani et al., 2014) included *Penicillium, Cladosporium*, and *Aspergillus* species which were tested for proteolytic, amylolytic, cellulolytic, lipolytic activities in the presence of 10% NaCl. *P. canescens* AJ5 from the cited study, which corresponds to strains S2, S10, and S13 from this research, did not show any of the above-mentioned activities. Meanwhile, *P. canescens* S2, S10 and S13 were great producers of cellulases (average EI 6.00) and esterase (average EI 3.58), but also mediocre lipase (average EI 1.88) and amylase (average EI 1.82), on a medium containing 5% NaCl. Other corresponding strains were *C. cladosporoides* JA14, JA18 (Jaouani et al., 2014) and FF11, S5 (this study), where strains presented in this article showed high amylolytic, cellulolytic, and lipolytic activity compared to no activity for sebkha isolates. Although none of the *Aspergillus* strains corresponded on species level, some genus-wide correlations can be made. While strains isolated from Sebkha El Melah did not produce any of the listed enzymes, *Aspergillus* strains isolated in Poland and Italy were significant producers of cellulase (average EI 4.99) and produced also amylase and lipase.

Another research involving halophilic fungi isolated from the Great Sebkha of Oran in Algeria (Chamekh et al., 2019) showed enzymatic activities but on a medium without salt. The study included *Fusarium equiseti* D3, which corresponds to FF2 strain from this work, and *Penicillium canescens* S18 affined to S2 and S13 strains (this study). In the case of both *F. equiseti* and *P. canescens*, they showed similar enzymatic profiles, but for cellulases Polish and Italian strains isolated in this research had 2 or 3 times higher enzymatic activity, respectively. Same for the amylolytic activity for S18 was only 0.15, while for S2, S10, and S13 EI was on average 1.76.

Obtained results could be also compared for corresponding strains obtained from non-saline conditions. Coronado-Ruiz et al. (2018) tested cellulolytic activity of fungi isolated from nineteenth century French drawings, among others *P. dimorphosporum* (isolate #5), *C. cladosporoides* (isolate #9 and #16) and *A. niger* (isolate #8 and #22). Also in this case, enzymatic activities were twice higher (*C. cladosporoides*—average EI for non-halophilic isolates of 1,54, while EI_FF11_ = 2.77 and EI_S5_ = 3.07) or even four times higher (*A. niger*—EI_#8_ = 0.92 and EI_#22_ = 1.086 comparing to EI_FF14_ = 4.37). Enzymatic Index for cellulolytic and xylanolytic activity was also checked for thermophilic fungal stains from dry soils of Telangana in India (Saroj et al., 2018). Nine *Aspergillus* strains from the presented study showed cellulolytic EI ranging from 1.02 to 1.5 and xylanolytic EI of 1.01 to 1.18. Meanwhile, five *Aspergillus* spp. strains from this study (FF7, FF8, FF10, and S7) had an average EI of cellulase of 4.99 and EI of xylanase of 1.63, showing significantly higher enzymatic activities of halophilic strains.

During this research, the potential of biosurfactant production was tested. *Acremonium sclerotigenum* FF3, an endophytic strain isolated from the root of *Atriplex* L. growing on saline soil in the area of Grosseto in Italy, showed the best hemolytic activity on medium with 10% (w/v) NaCl and thus was selected for further studies. *Acremonium sclerotigenum* was previously isolated from saline marine environments (Jalili et al., 2020) and was known as an endophyte (Lo Piccolo et al., 2015; Jalili et al., 2020; Yao et al., 2023). *A. sclerotigenum* has already been reported as a hydrophobin producer (Cicatiello et al., 2016, 2017; Pitocchi et al., 2023). Hydrophobins are small protein biosurfactants found in filamentous fungi known for their high surface activity (Vigil et al., 2024).

The evaluation of mycotoxin production in fungal strains is an important step in assessing their suitability for industrial and environmental applications, especially in potential food and feed industries (Bennett and Klich, 2003; Janik et al., 2021). Exposure to mycotoxins may lead to serious health problems such as a weakened immune system, vomiting, infertility, stomach issues, and even cancer (Liu et al., 2022). Many of tested filamentous fungi, including those with promising enzymatic capabilities, are also known producers of toxic secondary metabolites, such as aflatoxins, ochratoxins, and fumonisins. This especially refers to strains belonging to *Fusarium, Aspergillus* and *Penicillium* (Sweeney and Dobson, 1998; Greeff-Laubscher et al., 2019). Of all the tested strains, only one, *Penicillium canescens* S10, produced mycotoxins, specifically ochratoxin A. Although there is no available data on *P. canescens* producing this mycotoxin, ochratoxin A (OTA) is known to be produced by several strains of *Aspergillus* and *Penicillium*, mostly *P. verrucosum, P. nordicum*, and *P. expansum* (Cabañes et al., 2010; Gil-Serna et al., 2018).

## 5 Conclusions

This research shows that the fungal strains isolated from saline soils from contrasting pedoclimates have high enzymatic activities in 5% NaCl. This suggests that these fungi have adapted to the salinity conditions, which are the optimal growth conditions for most strains, and that increased environmental salinity does not inhibit their enzyme production. The unusually high activities of hydrolytic haloenzymes, compared to studies where fungi were obtained from non-saline conditions, indicate the great potential of the halophilic fungi presented in this article. They can have a wide range of industrial applications, also in the food industry, due to their limited production of the most common mycotoxins. Further research is necessary to discover the metabolic potential of mentioned halophilic fungal strains, but also to test and optimize production of enzymes found in this study. Special attention should be given to *Ramularia mali* FF1, which was the best producer of tested hydrolytic enzymes and had not been previously described for saline soil environments. Additionally, future studies should focus on investigating how varying NaCl concentrations influence enzymatic activity, biosurfactant production, and secondary metabolite synthesis. Such insights could deepen our understanding of fungal adaptation to salinity and enhance their potential us in biotechnological applications in saline conditions.

## Data Availability

The original contributions presented in the study are publicly available. This data can be found here: https://www.ncbi.nlm.nih.gov, accession numbers PQ596426-PQ596437; PP869091-PP869099; PQ644613-PQ644624.
